# Durable response of tislelizumab plus cisplatin, nab-paclitaxel followed by concurrent chemoradiotherapy in locoregionally advanced nasopharyngeal carcinoma: A case report

**DOI:** 10.1097/MD.0000000000032924

**Published:** 2023-02-17

**Authors:** Haifeng Tang, Donghong Yang, Guoqing Luo, Jiaqi He, Guihua Yi, Zihong Chen, Haiwen Li, Qianbing Luo, Ningxin Huang, Haiqing Luo

**Affiliations:** a Specialty of Head and Neck Oncology at Cancer Hospital of the Affiliated Hospital of Guangdong Medical University, Zhanjiang, Guangdong, China; b Department of Otorhinolaryngology Head and Neck Surgery at the Affiliated Hospital of Guangdong Medical University, Zhanjiang, Guangdong, China.

**Keywords:** cisplatin, immunotherapy, nab-paclitaxel, nasopharyngeal carcinoma, tislelizumab

## Abstract

Limited patients with locoregionally advanced nasopharyngeal carcinoma (LA-NPC) have achieved complete response (CR) from induction chemotherapy (IC). Neoadjuvant immunotherapy combined with chemotherapy has marked therapeutic effects in some locoregionally advanced solid tumors. However, its efficacy and safety of NPC have not been reported so far. The rapid response of neoadjuvant tislelizumab combined with chemotherapy on LA-NPC may be associated with long-term survival benefit.

**Patient concerns:**

A 57-year-old male patient presented with a 2-month history of bloody nasal discharge and right neck mass for 2 weeks.

**Diagnosis:**

The patient was eventually diagnosed with nasopharyngeal nonkeratinizing undifferentiated cell carcinoma (stage IVA).

**Interventions:**

The patient received tislelizumab combined with nanoparticle albumin-bound paclitaxel (nab-paclitaxel) nab-paclitaxel plus cisplatin for 4 cycles, followed by cisplatin-based concurrent chemoradiotherapy (CCRT).

**Outcomes:**

A partial response (PR) was achieved after 2 cycles of tislelizumab and nab-paclitaxel plus cisplatin, and CR was achieved after 4 cycles of neoadjuvant treatment. The duration of response lasted 24 months, and the patient was still in CR as of November 2022. The patient had no serious adverse event (AEs) during the treatment.

**Lessons:**

This case report showed that tislelizumab combined with cisplatin plus nab-paclitaxel followed CCRT for treatment of patients with LA-NPC may receive a fast and durable response with a manageable safety profile and long-term survival.

## 1. Introduction

Nasopharyngeal carcinoma (NPC) is one of the most common head and neck cancers prevalent in southeast Asia and southern China.^[1]^ More than 70% of patients were diagnosed with locoregionally advanced NPC (LA-NPC) at presentation.^[2]^ Although induction chemotherapy (IC) combined with concurrent chemoradiotherapy (CCRT) is the standard treatment for LA-NPC, with a 5-year overall survival (OS) rate of about 80% and 5-year distant metastasis rate of about 20%,^[3]^ new treatments are still needed to improve survival and outcomes for patients with LA-NPC.

Currently, nanoparticle albumin-bound paclitaxel (nab-paclitaxel) combined with cisplatin is a recommended treatment regimen for recurrent or metastatic NPC, but studies on the treatment of LA-NPC are limited. Nab-paclitaxel plus cisplatin serves as the first-line treatment for recurrent or metastatic NPC, and it may have a curative effect on LA-NPC. Recently, antibodies targeting programmed cell death 1 (PD-1) and programmed cell death ligand 1 (PD-L1) have been increasingly developed in tumor immunotherapy.^[4]^ Anti-PD-1 therapies with monoclonal antibodies have been approved for the treatment of recurrent or metastatic NPC.^[5–7]^ However, reports on immunotherapy as neoadjuvant therapy for LA-NPC are few. The therapeutic efficacy of anti-PD-1 monoclonal antibodies combined with IC in patients with LA-NPC remains unclear, and related clinical trials are ongoing (NCT04833257, NCT03700476). Some trials have shown that the objective efficacy rate of tislelizumab in the treatment of patients with recurrent metastatic NPC is 43%, which is higher than that of other anti-PD-1 monoclonal antibodies.^[8]^ Thus, tislelizumab is a highly promising new treatment for patients with LA-NPC.

Here, we report a patient with LA-NPC who received tislelizumab combined with nab-paclitaxel plus cisplatin chemotherapy for 4 cycles, followed by CCRT combined with tislelizumab. A complete response (CR) was then maintained by the patient after neoadjuvant immunotherapy plus chemotherapy. The patient has remained in CR for longer than 24 months at the time of filing this report.

## 2. Case presentation

The patient was a 57-year-old man without any other associated disease who presented with a 2-month history of bloody nasal discharge and right neck mass for 2 weeks. After a series of examinations including histopathological biopsy and magnetic resonance imaging (MRI), the patient was diagnosed with NPC. The TNM stage was T2N3M0 (stage IVA) according to the 8th edition of the American Joint Committee on Cancer staging system.

**Figure 1.**
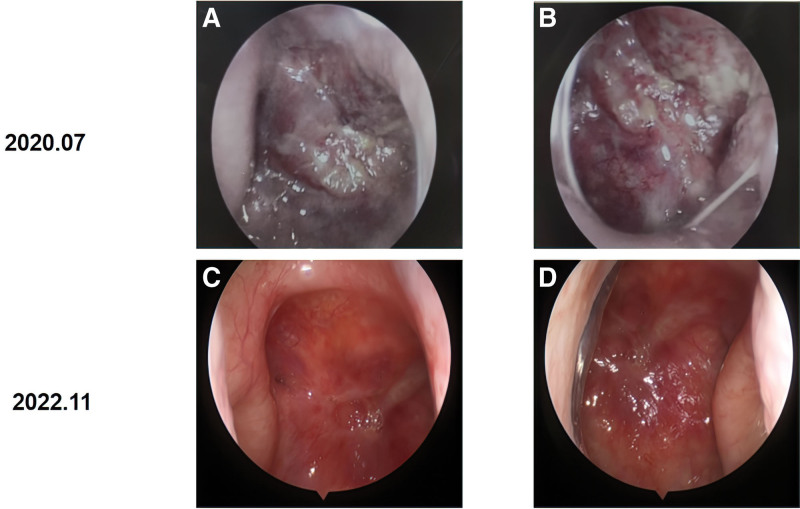
(A and B) Nasopharyngeal endoscopy performed the primary focus of NPC. Some fleshy masses and moderate could be seen on the posterior wall of the nasopharynx and pharyngeal recess. (C and D) Nasopharyngeal mucosa showed mild hyperemia and no obvious neoplasm was observed after chemotherapy and radiotherapy. NPC = nasopharyngeal carcinoma.

**Figure 2.**
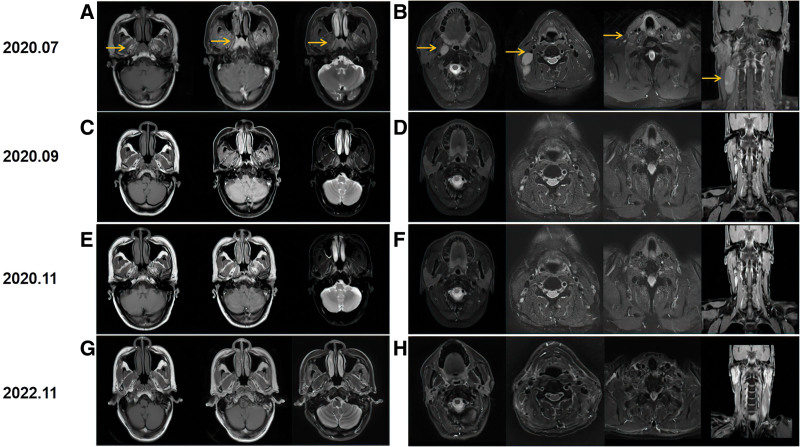
(A and B) MRI of the nasopharynx and lymph node before treatment (August, 2020). (C and D) MRI of the nasopharynx and lymph node after 2 cycles of treatment (September, 2020). (E and F) MRI of the nasopharynx and lymph node after 4 cycles of treatment (November, 2020). (G and H) MRI of the nasopharynx and lymph node about 24 months after treatment (November, 2022). MRI = magnetic resonance imaging.

**Figure 3.**
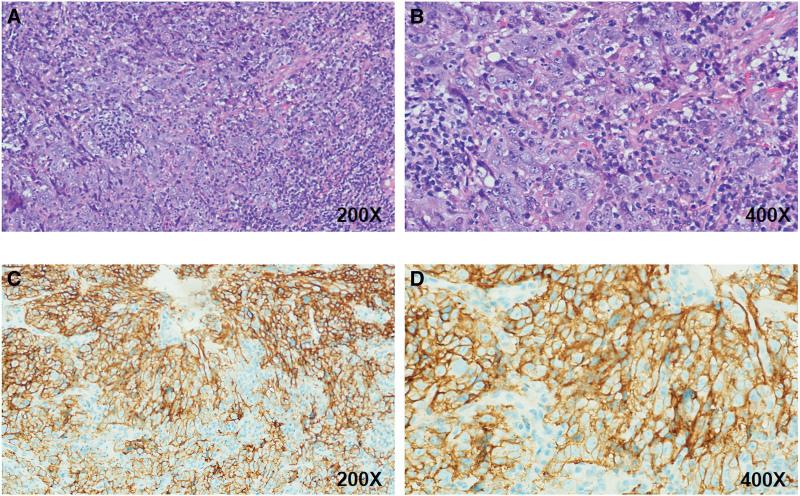
(A and B) The pathologic findings of the nasopharyngeal mass were nonkeratinized undifferentiated nasopharyngeal carcinoma. In the interstitial tissue, there are atypical cells with lamellar distribution, epithelioid, syncytial, vacuolar nuclei, and obvious nucleoli. (C and D) Immunohistochemical staining for PD-L1 expression (approximately 90% positive). PD-L1 = programmed cell death ligand 1.

Nasal endoscopy indicated some fleshy masses and moderate hyperemia in the posterior wall of nasopharynx and pharyngeal recess (Fig. 1A and B). MRI of the nasopharynx and neck examination detected nasopharyngeal lesions and enlarged bilateral cervical lymph nodes (Fig. 2A and B). Epstein–Barr virus (EBV) DNA copy number in the plasma of the patient was 87 copies/mL before starting therapy. Whole-body bone scan showed no signs of tumor metastasis in the whole-body bones. All additional laboratory data obtained from blood routine examination and biochemical blood tests of the patient were within the normal range. Biopsies obtained from the nasopharynx were analyzed to determine a diagnosis of nasopharyngeal nonkeratinizing undifferentiated cell carcinoma. The patient had detectable PD-L1-positive tumor expression (approximately 90% positive) in immunohistochemistry analysis (Fig. 3).

**Figure 4.**
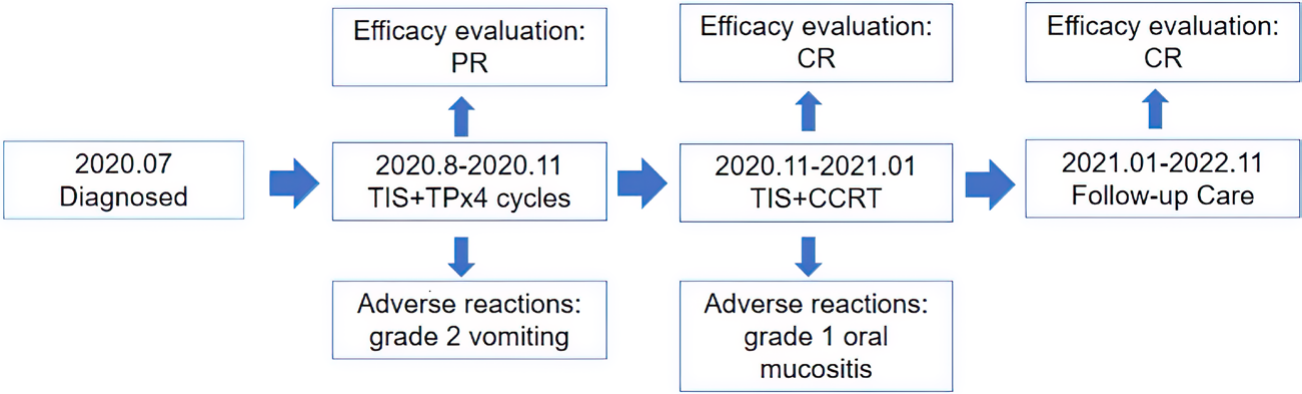
Timeline during the treatment of the patient. TIS = tislelizumab, TP = nab-paclitaxel and cisplatin.

Following the diagnosis, the patient received tislelizumab (200 mg) combined with nab-paclitaxel (260 mg/m^2^) plus cisplatin (80 mg/m^2^) every 3 weeks starting on August 4, 2020, for 2 cycles. Thereafter, the patient was assessed to have a partial response (PR) according to Response Evaluation Criteria in Solid Tumors (version1.1). The EBV DNA copy number in the plasma of the patient was <10 copies/mL. The patient continued to be treated with tislelizumab (200mg) combined with nab-paclitaxel (260 mg/m^2^) plus cisplatin (80 mg/m^2^) every 3 weeks starting on September 25, 2020, for 2 cycles. CR was achieved by the patient after 4 cycles of IC plus tislelizumab, and the EBV-DNA copy number of this patient was still <10 copies/mL. Soon afterwards, the patient received intensity-modulated radiation therapy from November 2020 to December 2020. Radiation doses were 69.96 Gy to gross tumor volume of the nasopharynx, 69.96 Gy to gross cervical lymph nodes, 62 Gy to the planning target volume 1, and 54 Gy to the planning target volume 2 in 33 fractions, 5 times per week. In the meantime, the patient received tislelizumab (200 mg) plus cisplatin (100 mg/m^2^) for 2 cycles. Subsequently, the tumor response was CR. Furthermore, the EBV-DNA copy number of this patient was still <10 copies/mL after CCRT, and the value remained unchanged until the last review. The patient experienced grade 2 vomiting during IC and grade 1 oral mucositis during CCRT. After the above treatment, MRI of his nasopharynx and neck examination showed CR (Fig. 2E–H). To date, the patient remains healthy. The timeline of treatment for the patient is depicted in Figure 4. Written informed consent was obtained from the patient for the publication of this case report.

## 3. Discussion

At present, the comprehensive therapy of the combination of IC and CCRT is the main treatment for LA-NPC. However, the bottleneck stage has been reached in the treatment of LA-NPC. To improve the efficacy of treatment, treatments with good efficacy and few side effects are needed for patients with LA-NPC.

Neoadjuvant chemotherapy followed by cisplatin-based CCRT is currently the main treatment option for LA-NPC.^[9]^ Neoadjuvant chemotherapy is commonly administered in locally advanced cancer to induce tumor shrinkage and eliminate NPC micro-metastasis to facilitate subsequent therapy. In addition, IC combined with CCRT can reduce the presence of metastasis and improve patient survival and prognosis.^[10]^ Ibassin-Majed et al included 20 randomized controlled trial studies with a total of 5144 patients for meta-analysis.^[11]^ Patients were divided into 7 subgroups according to chemotherapy and radiotherapy. The results demonstrated that the subgroup of IC combined with CCRT was superior to the other subgroups in the control rate of distant metastasis and ranked second in improving the tumor-free survival rate. Although IC combined with CCRT is considered an effective therapeutic modality for LA-NPC, the optimal IC remains to be defined.

Zhang et al reported that IC with the gemcitabine and cisplatin (GP) regimen added to chemoradiotherapy significantly improves OS and recurrence-free survival in patients with LA-NPC compared with chemoradiotherapy alone.^[12]^ However, the incidence of grade 3 or 4 acute adverse events (AEs) in the IC group was 75.7%, with a high incidence of bone marrow suppression, nausea, and vomiting. Frikha et al reported that the docetaxel, cisplatin and fluorouracil (TPF) regimen had good compliance with the treatment of LA-NPC, but the side effects were obvious, such as nausea/ vomiting, leukopenia and so on.^[13]^ The data presented in a meta-analysis suggested that the docetaxel and cisplatin regimen is the best IC regimen for OS and local recurrence-free survival in LA-NPC.^[14]^ The docetaxel and cisplatin regimen for 2 cycles followed by weekly CCRT appeared to be the most effective strategy for improving the median OS of patients with LA-NPC. Most patients experienced mild or moderate AEs, with no serious unexpected AEs suspected. However, traditional solvent-based paclitaxel is highly hydrophobic, so it is formulated with a mixture of polyol castor oil to facilitate intravenous infusion.^[15]^ The solvents increase the incidence of hypersensitivity and toxicity during injection. Therefore, patients usually need to receive pretreatments such as corticosteroids and antihistamine therapy before chemotherapy, which makes the actual clinical procedures more complicated and increases the risk of treatment for patients.^[16]^

Nab-paclitaxel, a complex of albumin and paclitaxel, was designed to overcome the above defects. It is readily soluble in normal saline for infusion. Albumin, as a carrier of paclitaxel, binds to the gp60 receptor on vascular endothelial cells of tumors and activates caveolin-1 on the cell membrane. With the help of caveolin-1, nab-paclitaxel is transported by endocytosis from the bloodstream into the tissue space. Albumin accumulating in tumor tissue increases the distribution of paclitaxel in tumor cells and improves the antitumor effects of paclitaxel.^[17]^ After 2 cycles of IC with nab-paclitaxel plus cisplatin and CCRT, the objective response rates were 97.2% and 100%, respectively, for patients with LA-NPC according to Liang et al.^[18]^ Thrombocytopenia (34.3%) and leukopenia (28.6%) were the most common grade 3 to 4 acute AEs throughout treatment. Therefore, the IC regimen of nab-paclitaxel plus cisplatin is available for patients with LA-NPC due to mild side effects and significant antitumor effects. However, for LA-NPC patients with lymph node stage N3, the optimal number of induction cycles keeps still controversial. Some studies reported that patients with stage N3, who have high risk of recurrence or metastasis, and receiving 4 cycles of IC might reduce the risk of recurrence or metastasis and improve the survival rate.^[19,20]^

Recently, PD-1/PD-L1 inhibitors have been increasingly used in cancer therapy. PD-L1 is an immunoinhibitory molecule, which induces T-cell-mediated immune tolerance by activating PD-1 located on the surface of T cells.^[21]^ Thus, a significant antitumor effect has been shown by immune therapies that target the PD-1/PD-L1 axis in certain types of solid tumors, including melanoma, non-small cell lung cancer (NSCLC), head and neck carcinomas and so on. Cancer immunotherapy is now recognized as another important cancer treatment besides surgery, chemotherapy, and radiotherapy. Some studies confirmed that antibodies targeting PD-1 showed promising efficacy in the treatment of NSCLC,^[22–26]^ recurrent or metastatic NPC,^[5–7]^ esophageal cancer,^[27,28]^ recurrent or metastatic head and neck squamous cell carcinoma,^[29]^ early triple-negative breast cancer,^[30,31]^ etc. Some clinical trials reported that in the patients with metastatic squamous non-small cell lung cancer treated, compared with chemotherapy alone, immunotherapy combined with chemotherapy could significantly prolong OS and progression-free survival (PFS).^[32–34]^ Zhang et al found that PFS is significantly prolonged in patients with recurrent or metastatic NPC who receive camrelizumab plus GP compared with placebo plus GP.^[6]^ The safety profiles of camrelizumab plus chemotherapy are manageable. A multicenter randomized phase 3 trial demonstrated the efficacy and safety of toripalimab plus chemotherapy as first-line treatment in advanced NPC. The results demonstrated that toripalimab plus GP prolongs the PFS compared with GP alone in recurrent or metastatic NPC but maintains a manageable safety profile.^[7]^ The current NCCN guidelines recommend anti-PD-1 monoclonal antibody as a subsequent-line treatment for recurrent or metastatic NPC.^[35]^ However, side effects associated with PD-1/PD-L1 blockade have been characterized by a distinct range of toxic effects, termed immune-related AEs, including rash, colitis, hepatitis, and hypothyroidism.

Tislelizumab is a humanized monoclonal antibody with high affinity and specificity for PD-1 that was specifically engineered to minimize FcɣR binding on macrophages to abrogate antibody-dependent phagocytosis, which is a potential mechanism of T-cell clearance and resistance to anti-PD-1 therapy.^[36]^ Reports from a early phase study showed that single-agent tislelizumab is generally well tolerated and demonstrates antitumor activity in patients with advanced refractory solid tumors, including NPC and NSCLC.^[37]^ Some studies have shown that tislelizumab was a rational choice in the treatment of patients with recurrent and metastatic NPC, with an objective response rate of 43%.^[8]^

At present, promising outcomes have been revealed by immunotherapy combined with radiotherapy in several types of malignancies, including NSCLC,^[38]^ recurrent NPC,^[39]^ etc. A clinical trial reported that toripalimab combined with intensity-modulated radiation therapy is tolerable and shows promising antitumor activity in patients with recurrent NPC.^[39]^ Some studies on antibodies targeting PD-1 combined with radiotherapy in the treatment of LA-NPC are still in progress (NCT05097209, NCT03700476). Additionally, the synergistic effects on local and distant tumor control have been demonstrated by several studies when radiation therapy was added to immunotherapy.^[40]^ In this case, the patient with stage IVA NPC was treated with tislelizumab combined with IC and CCRT. The patient received neoadjuvant immunotherapy plus chemotherapy and achieved CR. Immunotherapy combined with chemotherapy can regulate the number of immune cells and inhibit the immune escape mechanism of tumors.^[41]^ Besides, immunotherapy combined with radiotherapy has a synergistic effect on tumors. Radiotherapy can not only destroy malignant cells but also change the immune phenotype of residual tumor cells, such as upregulating the tumor expression of PD-L1. Radiotherapy can enhance the release of tumor antigens and improve the immune response. Moreover, the patient received IC of nab-paclitaxel combined with cisplatin. Nab-paclitaxel has a good curative effect and almost eliminates the risk of allergic reactions to solvent-based paclitaxel. Meanwhile, nab-paclitaxel is significantly effective and less toxic than solvent-based paclitaxel in antitumor activity, because of high tumor tissue distribution and restricted distribution in normal tissues. Albumin addresses the solubility and solution stability of paclitaxel. Surprisingly, after 2 cycles of tislelizumab plus IC, the tumor response was PR; after 4 cycles of IC plus tislelizumab, the tumor response was CR. Meanwhile, the tumor response of CR was maintained during follow-up after treatment for 24 months. Notably, compared with other patients with LA-NPC, this patient achieved CR after IC for 4 cycles, which was relatively rare. When the patient has CR after neoadjuvant immunotherapy plus chemotherapy, can we replan target delineation and dose prescription in subsequent radiotherapy? This is an issue worth discussing and requires further research. Regrettably, the sample size in this work was small, so large-scale clinical trials need to be performed to verify the generalizability of the present conclusion.

In summary, neoadjuvant immunotherapy plus chemotherapy followed by CCRT had a good effect in the treatment of the patient with LA-NPC in this case, and it was safe and well tolerated for the patient with LA-NPC.

## 4. Conclusion

In this case, the patient with stage IVA NPC treated with neoadjuvant immunotherapy plus chemotherapy followed by CCRT showed meaningful therapeutic efficacy and a manageable safety profile. The rapid response of this neoadjuvant treatment may be associated with long-term survival benefit. This case can be regarded as a reference for the treatment of patients with the same disease. In the future, we plan to conduct similar clinical trials with larger clinical sizes.

## Author contributions

**Conceptualization:** Haifeng Tang, Donghong Yang.

**Data curation:** Zihong Chen, Haiwen Li, Qianbing Luo, Ningxin Huang.

**Formal analysis:** Haifeng Tang, Guoqing Luo, Jiaqi He, Guihua Yi, Haiqing Luo.

**Funding acquisition:** Haiqing Luo.

**Investigation:** Haifeng Tang, Donghong Yang, Jiaqi He.

**Methodology:** Haifeng Tang, Donghong Yang, Jiaqi He, Guihua Yi.

**Project administration:** Zihong Chen, Haiqing Luo.

**Resources:** Haifeng Tang, Donghong Yang, Guoqing Luo, Haiqing Luo.

**Supervision:** Haiqing Luo.

**Writing – original draft:** Haifeng Tang, Donghong Yang, Guoqing Luo.

**Writing – review & editing:** Haifeng Tang, Haiqing Luo.

## References

[1] Wei K Zheng R Zhang S. Nasopharyngeal carcinoma incidence and mortality in China, 2013. Chin J Cancer. 2017;36:90. 29122009 10.1186/s40880-017-0257-9 PMC5679327

[2] Chen YP Chan ATC Le QT. Nasopharyngeal carcinoma. Lancet. 2019;394:64–80. 31178151 10.1016/S0140-6736(19)30956-0

[3] Pan JJ Ng WT Zong JF. Proposal for the 8th edition of the AJCC/UICC staging system for nasopharyngeal cancer in the era of intensity-modulated radiotherapy. Cancer. 2016;122:546–58. 26588425 10.1002/cncr.29795 PMC4968037

[4] Ribas A Wolchok JD. Cancer immunotherapy using checkpoint blockade. Science. 2018;359:1350–5. 29567705 10.1126/science.aar4060 PMC7391259

[5] Mai H Chen Q Chen D. Toripalimab or placebo plus chemotherapy as first-line treatment in advanced nasopharyngeal carcinoma: a multicenter randomized phase 3 trial. Nat Med. 2021;27:1536–43. 34341578 10.1038/s41591-021-01444-0

[6] Yang Y Qu S Li J. Camrelizumab versus placebo in combination with gemcitabine and cisplatin as first-line treatment for recurrent or metastatic nasopharyngeal carcinoma (CAPTAIN-1st): a multicentre, randomised, double-blind, phase 3 trial. Lancet Oncol. 2021;22:1162–74. 34174189 10.1016/S1470-2045(21)00302-8

[7] Wang F Wei X Feng J. Efficacy, safety, and correlative biomarkers of toripalimab in previously treated recurrent or metastatic nasopharyngeal carcinoma: a phase II clinical trial (POLARIS-02). J Clin Oncol. 2021;39:704–12. 33492986 10.1200/JCO.20.02712 PMC8078488

[8] Shen L Guo J Zhang Q. Tislelizumab in Chinese patients with advanced solid tumors: an open-label, non-comparative, phase 1/2 study. J ImmunoTher Cancer. 2020;8:e000437e437. 32561638 10.1136/jitc-2019-000437 PMC7304812

[9] Wang B Xiao B Lin G. The efficacy and safety of induction chemotherapy combined with concurrent chemoradiotherapy versus concurrent chemoradiotherapy alone in nasopharyngeal carcinoma patients: a systematic review and meta-analysis. BMC Cancer. 2020;20:393. 32375701 10.1186/s12885-020-06912-3 PMC7204295

[10] Cao S Yang Q Guo L. Neoadjuvant chemotherapy followed by concurrent chemoradiotherapy versus concurrent chemoradiotherapy alone in locoregionally advanced nasopharyngeal carcinoma: a phase III multicentre randomised controlled trial. Eur J Cancer. 2017;75:14–23. 28214653 10.1016/j.ejca.2016.12.039

[11] Ribassin-Majed L Marguet S Lee AWM. What is the best treatment of locally advanced nasopharyngeal carcinoma? An individual patient data network meta-analysis. J Clin Oncol. 2017;35:498–505. 27918720 10.1200/JCO.2016.67.4119 PMC5791836

[12] Zhang Y Chen L Hu G. Gemcitabine and cisplatin induction chemotherapy in nasopharyngeal carcinoma. N Engl J Med. 2019;381:1124–35. 31150573 10.1056/NEJMoa1905287

[13] Frikha M Auperin A Tao Y. A randomized trial of induction docetaxel–cisplatin–5FU followed by concomitant cisplatin-RT versus concomitant cisplatin-RT in nasopharyngeal carcinoma (GORTEC 2006-02). Ann Oncol. 2018;29:731–6. 29236943 10.1093/annonc/mdx770

[14] Bongiovanni A Vagheggini A Fausti V. Induction chemotherapy plus concomitant chemoradiotherapy in nasopharyngeal carcinoma: an updated network meta-analysis. Crit Rev Oncol Hematol. 2021;160:103244. 33582249 10.1016/j.critrevonc.2021.103244

[15] Gelderblom H Verweij J Nooter K. Cremophor EL: the drawbacks and advantages of vehicle selection for drug formulation. Eur J Cancer. 2001;37:1590–8. 11527683 10.1016/s0959-8049(01)00171-x

[16] Marupudi NI Han JE Li KW. Paclitaxel: a review of adverse toxicities and novel delivery strategies. Expert Opin Drug Saf. 2007;6:609–21. 17877447 10.1517/14740338.6.5.609

[17] Desai N Trieu V Yao Z. Increased antitumor activity, intratumor paclitaxel concentrations, and endothelial cell transport of cremophor-free, albumin-bound paclitaxel, ABI-007, compared with cremophor-based paclitaxel. Clin Cancer Res. 2006;12:1317–24. 16489089 10.1158/1078-0432.CCR-05-1634

[18] Ke L Xia W Qiu W. A phase II trial of induction NAB-paclitaxel and cisplatin followed by concurrent chemoradiotherapy in patients with locally advanced nasopharyngeal carcinoma. Oral Oncol. 2017;70:7–13. 28622892 10.1016/j.oraloncology.2017.04.018

[19] Wei J Feng H Xiao W. Cycle number of neoadjuvant chemotherapy might influence survival of patients with T1-4N2-3M0 nasopharyngeal carcinoma. Chin J Cancer Res. 2018;30:51–60. 29545719 10.21147/j.issn.1000-9604.2018.01.06 PMC5842233

[20] Zhang Y Chen M Chen C. The efficacy and toxicities of intensive induction chemotherapy followed by concurrent chemoradiotherapy in nasopharyngeal carcinoma patients with N disease. Sci Rep. 2017;7:3668. 28623353 10.1038/s41598-017-03963-8 PMC5473919

[21] Sun C Mezzadra R Schumacher TN. Regulation and function of the PD-L1 checkpoint. Immunity. 2018;48:434–52. 29562194 10.1016/j.immuni.2018.03.014 PMC7116507

[22] Jotte R Cappuzzo F Vynnychenko I. Atezolizumab in combination with Carboplatin and Nab-Paclitaxel in advanced squamous non-small-cell lung cancer (IMpower131): results from a randomized phase III trial. J Thorac Oncol. 2020;15:1351–60. 32302702 10.1016/j.jtho.2020.03.028

[23] Sugawara S Lee JS Kang JH. Nivolumab with carboplatin, paclitaxel, and bevacizumab for first-line treatment of advanced nonsquamous non-small-cell lung cancer. Ann Oncol. 2021;32:1137–47. 34139272 10.1016/j.annonc.2021.06.004

[24] West H McCleod M Hussein M. Atezolizumab in combination with carboplatin plus nab-paclitaxel chemotherapy compared with chemotherapy alone as first-line treatment for metastatic non-squamous non-small-cell lung cancer (IMpower130): a multicentre, randomised, open-label, phase 3 trial. Lancet Oncol. 2019;20:924–37. 31122901 10.1016/S1470-2045(19)30167-6

[25] Paz-Ares L Ciuleanu TE Cobo M. First-line nivolumab plus ipilimumab combined with two cycles of chemotherapy in patients with non-small-cell lung cancer (CheckMate 9LA): an international, randomised, open-label, phase 3 trial. Lancet Oncol. 2021;22:198–211. 33476593 10.1016/S1470-2045(20)30641-0

[26] Lu S Wang J Yu Y. Tislelizumab plus chemotherapy as first-line treatment for locally advanced or metastatic nonsquamous NSCLC (RATIONALE 304): a randomized phase 3 trial. J Thorac Oncol. 2021;16:1512–22. 34033975 10.1016/j.jtho.2021.05.005

[27] Doki Y Ajani JA Kato K. Nivolumab combination therapy in advanced esophageal squamous-cell carcinoma. N Engl J Med. 2022;386:449–62. 35108470 10.1056/NEJMoa2111380

[28] Sun JM Shen L Shah MA. Pembrolizumab plus chemotherapy versus chemotherapy alone for first-line treatment of advanced oesophageal cancer (KEYNOTE-590): a randomised, placebo-controlled, phase 3 study. Lancet. 2021;398:759–71. 34454674 10.1016/S0140-6736(21)01234-4

[29] Burtness B Harrington KJ Greil R. Pembrolizumab alone or with chemotherapy versus cetuximab with chemotherapy for recurrent or metastatic squamous cell carcinoma of the head and neck (KEYNOTE-048): a randomised, open-label, phase 3 study. Lancet. 2019;394:1915–28. 31679945 10.1016/S0140-6736(19)32591-7

[30] Schmid P Cortes J Dent R. Event-free survival with Pembrolizumab in early triple-negative breast cancer. N Engl J Med. 2022;386:556–67. 35139274 10.1056/NEJMoa2112651

[31] Schmid P Cortes J Pusztai L. Pembrolizumab for early triple-negative breast cancer. N Engl J Med. 2020;382:810–21. 32101663 10.1056/NEJMoa1910549

[32] Paz-Ares L Luft A Vicente D. Pembrolizumab plus chemotherapy for squamous non-small-cell lung cancer. N Engl J Med. 2018;379:2040–51. 30280635 10.1056/NEJMoa1810865

[33] Socinski MA Jotte RM Cappuzzo F. Atezolizumab for first-line treatment of metastatic nonsquamous NSCLC. N Engl J Med. 2018;378:2288–301. 29863955 10.1056/NEJMoa1716948

[34] Gandhi L Rodríguez-Abreu D Gadgeel S. Pembrolizumab plus chemotherapy in metastatic non-small-cell lung cancer. N Engl J Med. 2018;378:2078–92. 29658856 10.1056/NEJMoa1801005

[35] Caudell JJ Gillison ML Maghami E. NCCN Guidelines® insights: head and neck cancers, Version 1.2022. J Natl Compr Canc Netw. 2022;20:224–34. 35276673 10.6004/jnccn.2022.0016

[36] Zhang T Song X Xu L. The binding of an anti-PD-1 antibody to FcγRΙ has a profound impact on its biological functions. Cancer Immunol Immunother. 2018;67:1079–90. 29687231 10.1007/s00262-018-2160-x PMC6006217

[37] Desai J Deva S Lee JS. Phase IA/IB study of single-agent tislelizumab, an investigational anti-PD-1 antibody, in solid tumors. J ImmunoTher Cancer. 2019;8:e453. 10.1136/jitc-2019-000453 PMC7295442 32540858

[38] Kordbacheh T Honeychurch J Blackhall F. Radiotherapy and anti-PD-1/PD-L1 combinations in lung cancer: building better translational research platforms. Ann Oncol. 2018;29:301–10. 29309540 10.1093/annonc/mdx790

[39] Hua Y You R Wang Z. Toripalimab plus intensity-modulated radiotherapy for recurrent nasopharyngeal carcinoma: an open-label single-arm, phase II trial. J ImmunoTher Cancer. 2021;9:e3290. 10.1136/jitc-2021-003290 PMC8593727 34782428

[40] Sharabi AB Lim M DeWeese TL. Radiation and checkpoint blockade immunotherapy: radiosensitisation and potential mechanisms of synergy. Lancet Oncol. 2015;16:e498–509. 26433823 10.1016/S1470-2045(15)00007-8

[41] Emens LA Middleton G. The interplay of immunotherapy and chemotherapy: harnessing potential synergies. Cancer Immunol Res. 2015;3:436–43. 25941355 10.1158/2326-6066.CIR-15-0064 PMC5012642

